# Supplementary material for: Health and disease markers correlate with gut microbiome composition across thousands of people
Source: Nat Commun. 2020 Oct 15;11:5206. doi: 10.1038/s41467-020-18871-1 (PMC7562722; doi:10.1038/s41467-020-18871-1)
Supplement: Supplementary file 1 — Supplementary Information [file 41467_2020_18871_MOESM1_ESM.pdf]

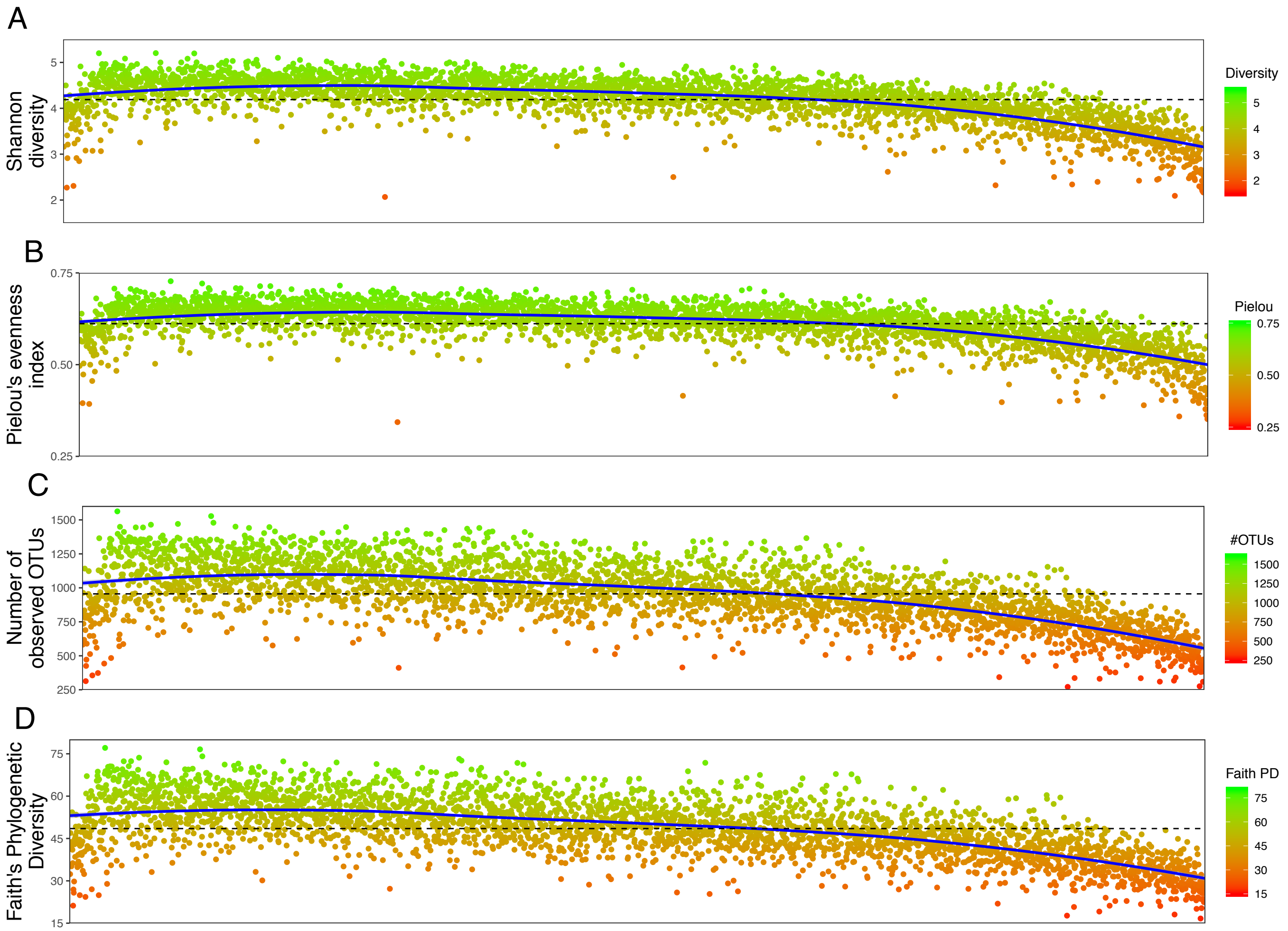

Supplementary Figure 1. Other microbial diversity measures agree with the pattern of Shannon diversity seen across the Bacteroidetes-to-Firmicutes axis. Shown are scatter plots of Shannon diversity index (**A**), Pielou's evenness index (**B**), number of observed OTUs (**C**), and Faith's Phylogenetic Distance index (**D**) across samples ordered by increasing abundance of Bacteroidetes (similar to ordering in **Figure 1**). Spearman's correlation coefficients with Shannon diversity were ρ=0.97, ρ=0.83, and ρ=0.86, for Pielou's index, observed OTUs, and Faith's index, respectively. N=3,409 biologically independent samples in all panels.

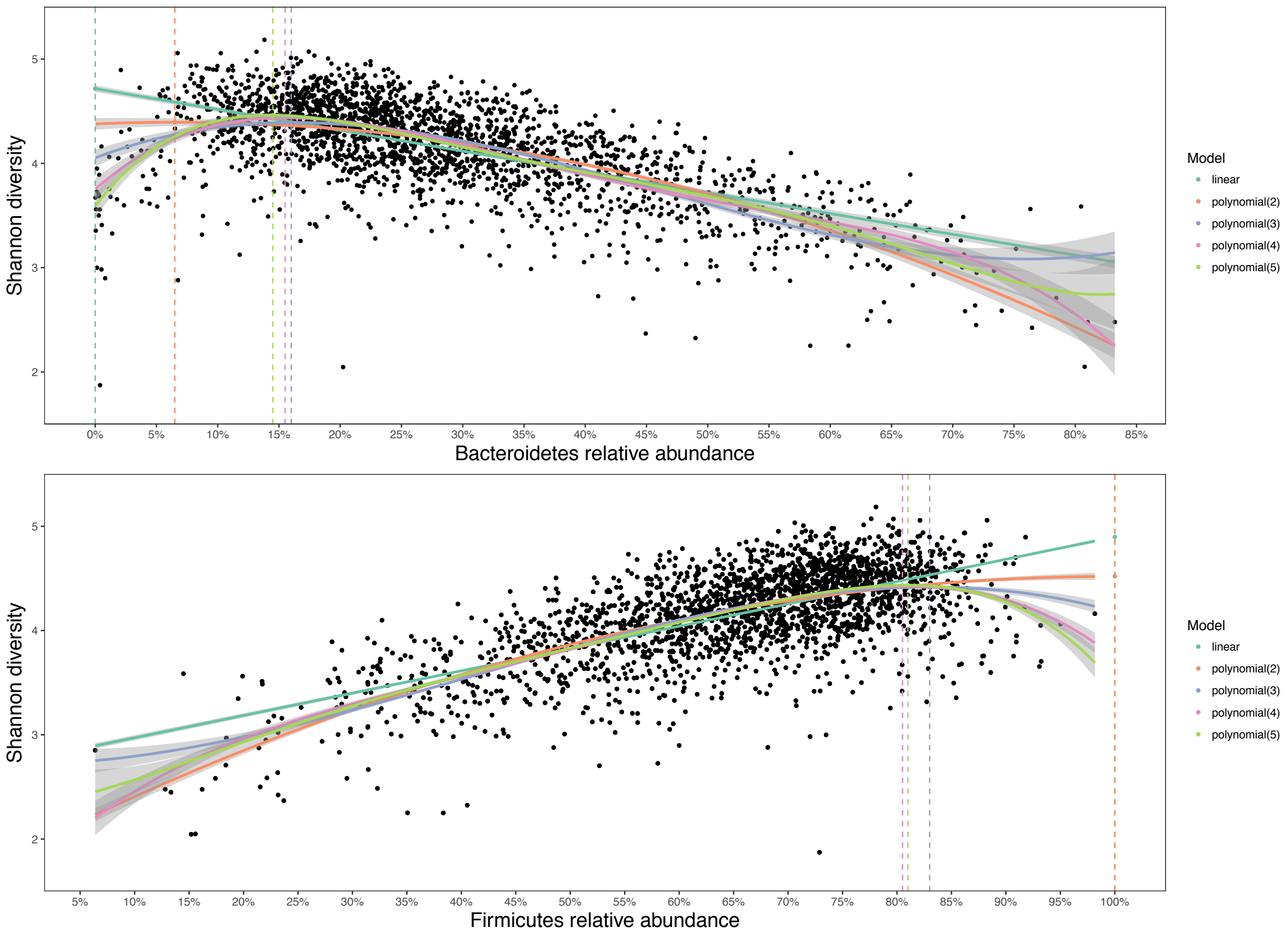

Supplementary Figure 2. The diversity "sweet-spot" on the Bacteroidetes-to-Firmicutes axis. Shown are scatter plots of the Shannon diversity index (y-axis) as a function of the relative abundance (x-axis) of Bacteroidetes (top panel) and Firmicutes (bottom panel). Curves represent polynomials of different degrees (1-5) fit to the data points, and the dashed lines represent the relative abundance with maximum diversity for each curve. Lines indicate the loess regression fit and the shaded area represents the 95% confidence interval. N=3,409 biologically independent samples in both panels.

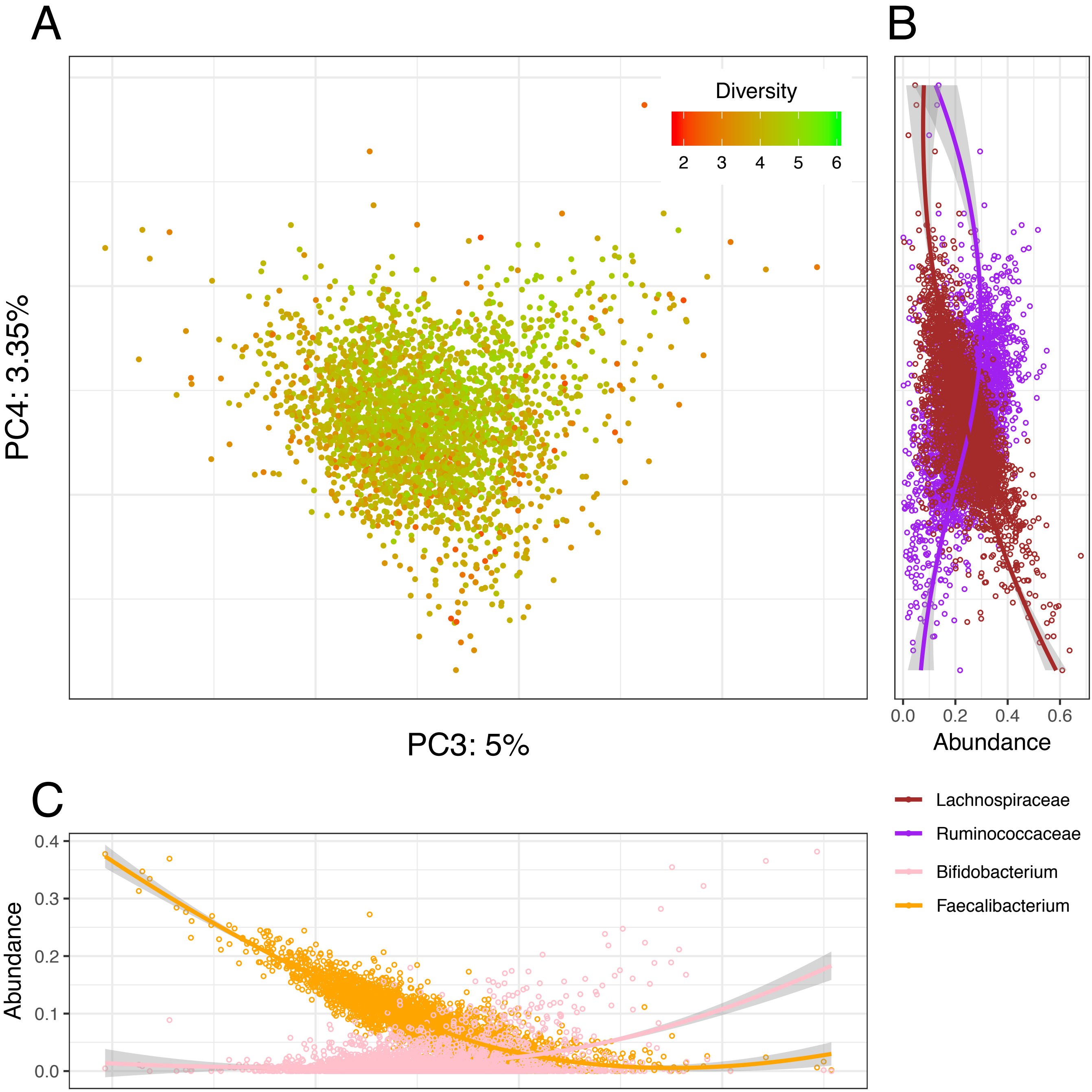

Supplementary Figure 3. Principal coordinate analysis (PCoA) of the taxonomic composition of the gut microbiome using the weighted UniFrac distance. (**A**) Shown is the principal coordinate analysis (PCoA) plot generated by applying PCoA to the weighted UniFrac distances between samples (as output of QIIME). Each point represents one sample, and the samples are colored by their Shannon diversity index. (**B+C**) Shown are scatter plots of the relative abundances of the phyla Bacteroidetes and Firmicutes (**B**) and the genera *Bacteroides* and *Prevotella* (**C**) across the corresponding principal component. For each sample in (**A**), there are 2 corresponding points in (**B**) and 2 corresponding points in (**C**). Lines indicate the loess regression fit and the shaded area represents the 95% confidence interval. N=3,409 biologically independent samples in all panels.

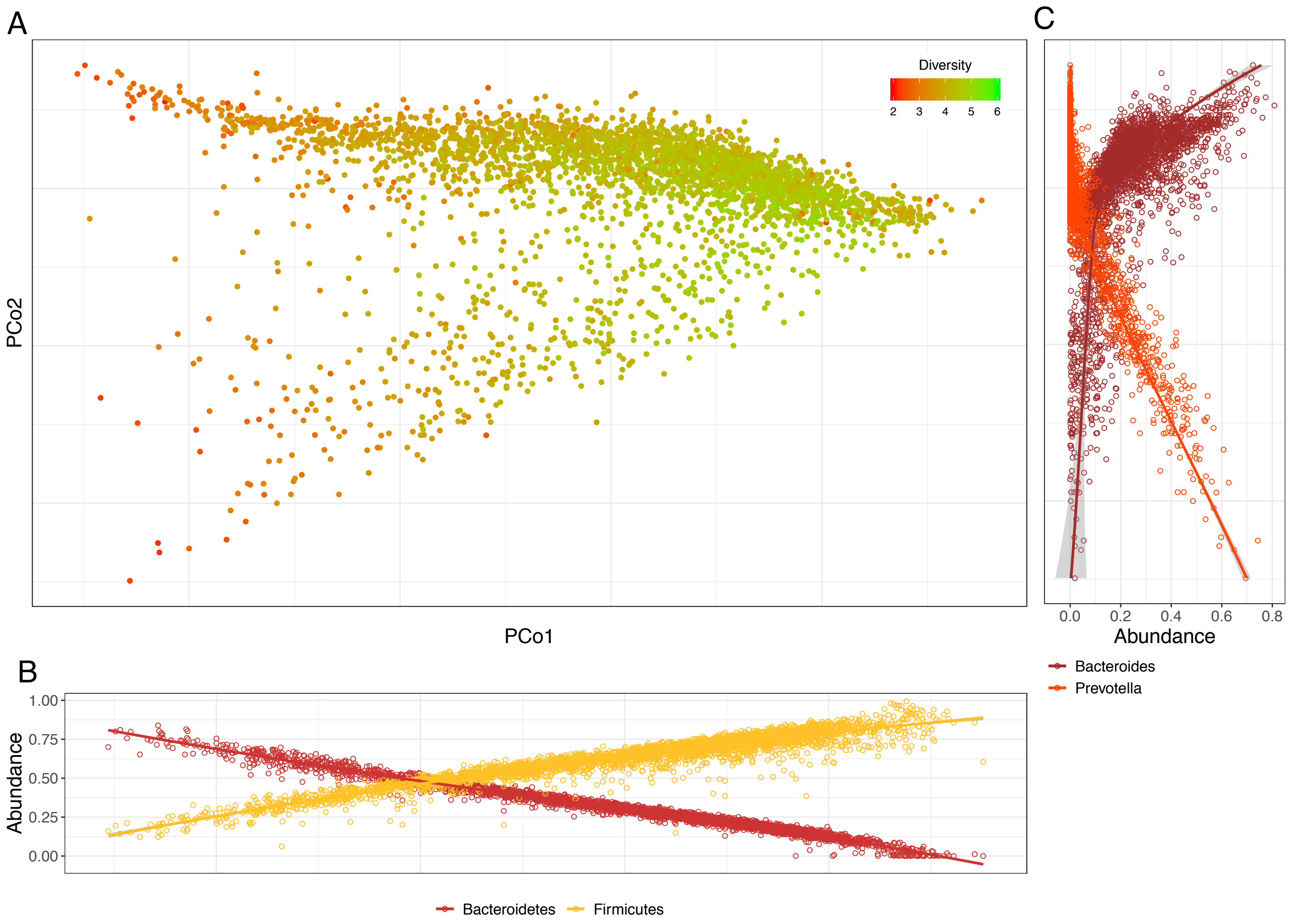

Supplementary Figure 4. Principal coordinate analysis (PCoA) of the taxonomic composition of the gut microbiome using the Bray-Curtis distance. (**A**) Shown is the principal coordinate analysis (PCoA) plot generated by applying PCoA to the Bray-Curtis distance between samples (using genus-level abundance). Each point represents one sample, and the samples are colored by their Shannon diversity index. (**B+C**) Shown are scatter plots of the relative abundances of the phyla Bacteroidetes and Firmicutes (**B**) and the genera *Bacteroides* and *Prevotella* (**C**) across the corresponding principal component. For each sample in (**A**), there are 2 corresponding points in (**B**) and 2 corresponding points in (**C**). Lines indicate the loess regression fit and the shaded area represents the 95% confidence interval. N=3,409 biologically independent samples in all panels.

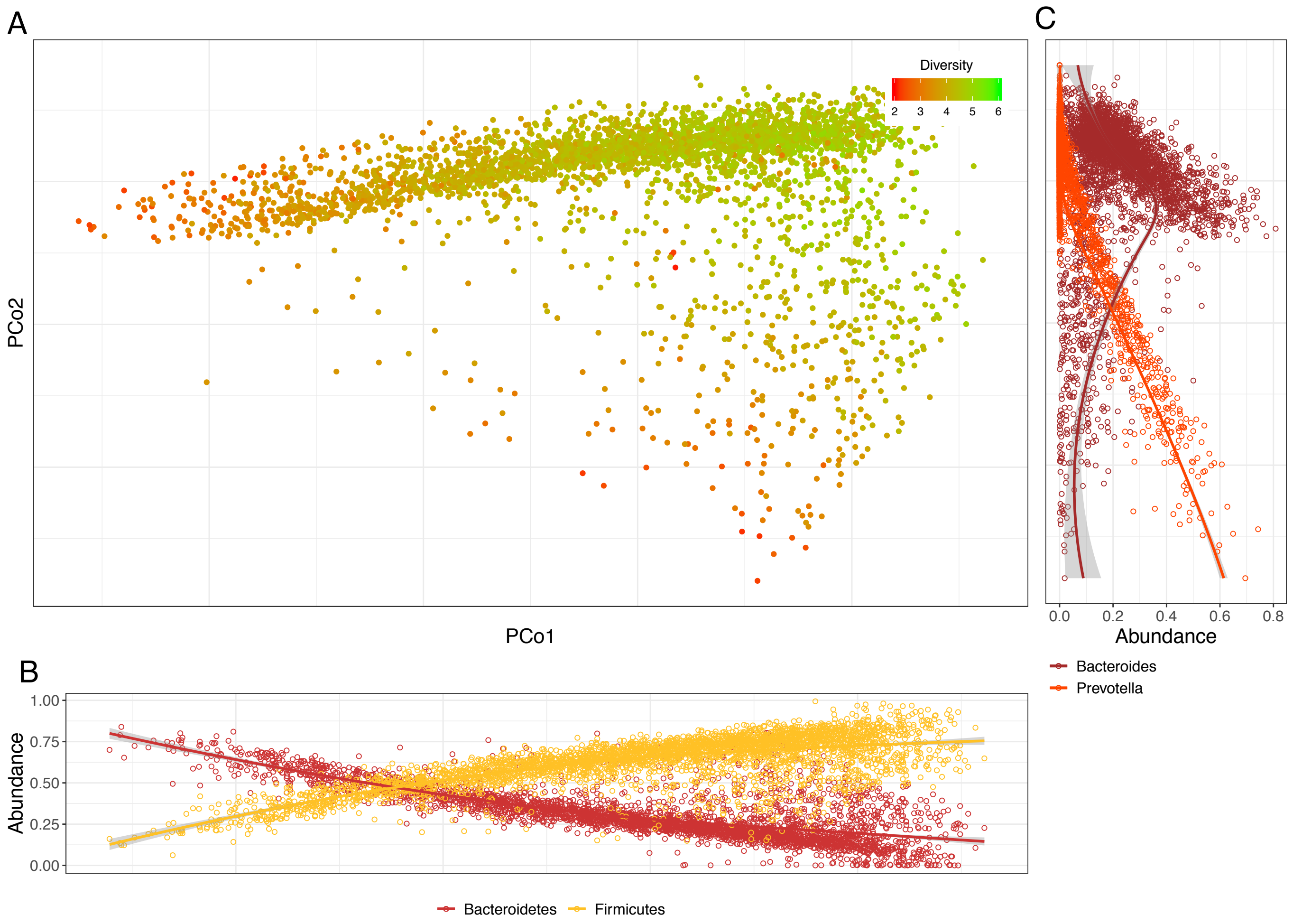

Supplementary Figure 5. Principal components 3 and 4 of the taxonomic composition of the gut microbiome. (**A**) Shown are the third and fourth principal components from the PCA generated by applying edgePCA to the OTU-level counts data. Each point represents one sample, and the samples are colored by their Shannon diversity index. The percent of variation explained by each principal component is depicted on each axis. (**B+C**) Shown are scatter plots of the relative abundances of the families *Lachnospiraceae* and *Ruminococcaceae* (**B**) and the genera *Bifodobacterium* and *Faecalibacterium* (**C**) across the corresponding principal component. For each sample in (**A**), there are 2 corresponding points in (**B**) and 2 corresponding points in (**C**). Lines indicate the loess regression fit and the shaded area represents the 95% confidence interval. N=3,409 biologically independent samples in all panels.

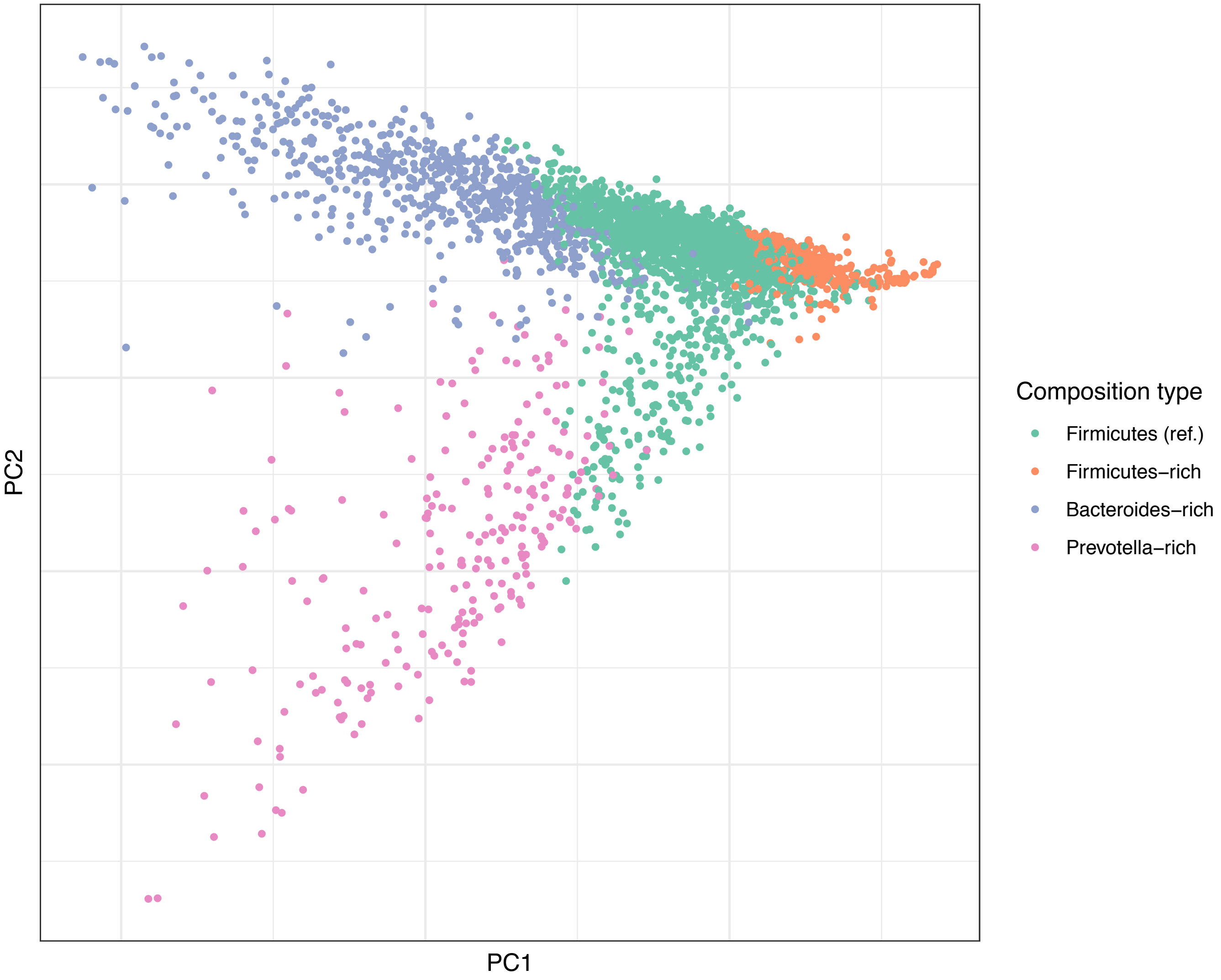

Supplementary Figure 6. Clusters of taxonomic composition. Shown is the principal component analysis (PCA) plot generated by applying edgePCA to the OTU-level counts data. Each point represents one sample, and the samples are colored by their defined taxonomic cluster. N=3,409 biologically independent samples.

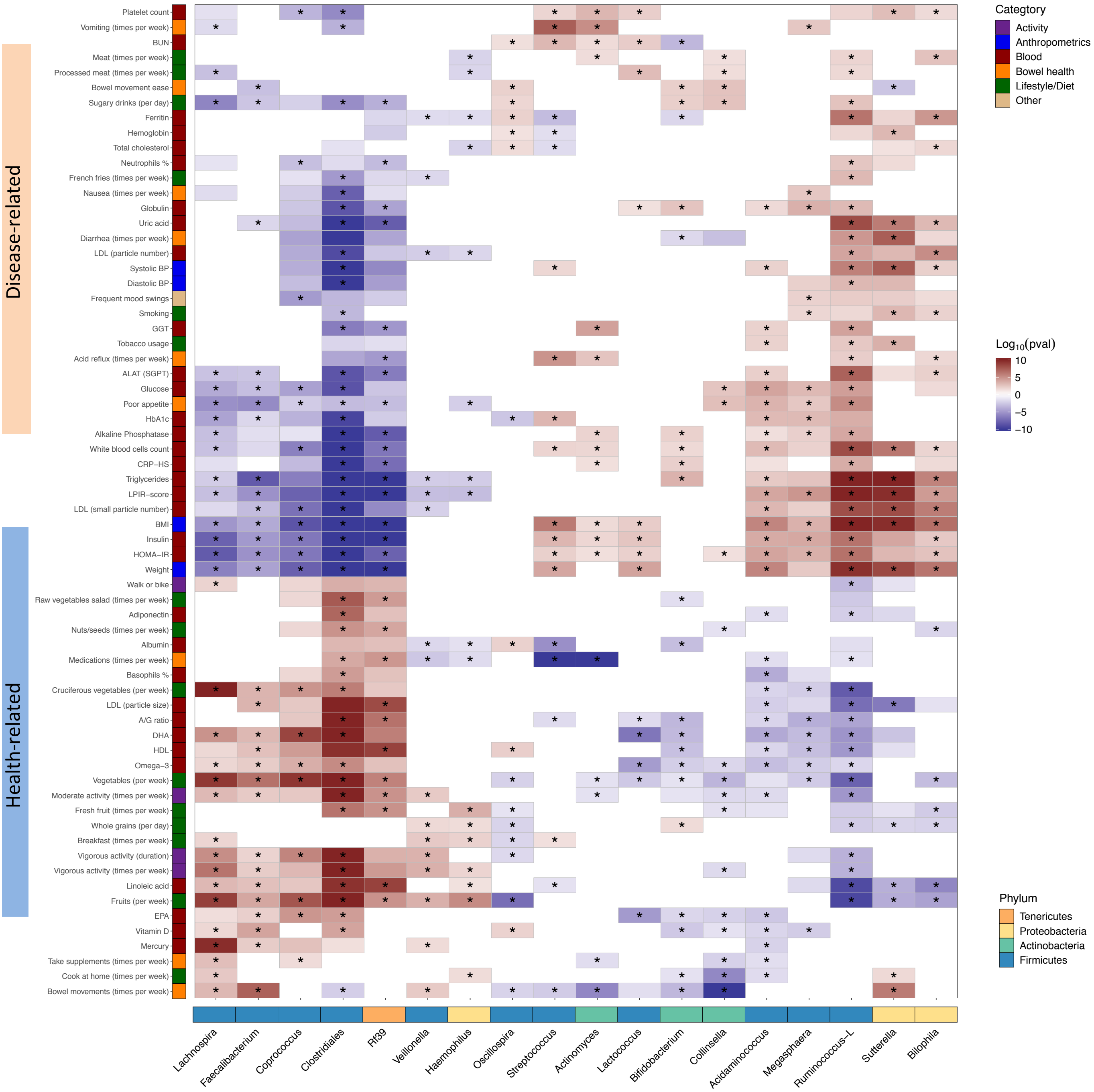

Supplementary Figure 7. Diversity-adjusted significant associations between microbial genera and multiple factors. Shown is a heatmap of the microbial genera (x-axis) that were found to be significantly associated with different factors (y-axis) using generalized linear models adjusted for confounding factors (see Methods). Only genera that had at least 20 *diversity-adjusted* significant associations are shown in the plot, along with factors that had *diversity-adjusted* significant associations with them (see full list of associations in **Supplementary Table 3**). For each analyte (e.g., lifestyle, diet, clinical test), associations were tested by fitting generalized linear models (see Methods). The significant heatmap cells (after correcting for multiple hypotheses with FDR-corrected p<0.05) are represented by the significance of the p-value (indicated by saturation, e.g., values of 10 or -10 indicate that p-value = 1^{-10}) and the direction of association (indicated by color, e.g., red is positively associated). Each factor is colored by the category to which it belongs, and each genus is colored by the phylum to which it belongs. Associations that are still significant after adjusting for microbiome diversity are marked with an Asterisk.
