# Supplementary material for: Health and disease markers correlate with gut microbiome composition across thousands of people
Source: Nat Commun. 2020 Oct 15;11:5206. doi: 10.1038/s41467-020-18871-1 (PMC7562722; doi:10.1038/s41467-020-18871-1)
Supplement: Supplementary file 3 — Description of Additional Supplementary Files [file 41467_2020_18871_MOESM3_ESM.pdf]

Description of Additional Supplementary Files

File Name: Supplementary Data 1-9
Description: Additional results derived from the various analyses described in the accompanying manuscript.
