# Supplementary material for: Health and disease markers correlate with gut microbiome composition across thousands of people
Source: Nat Commun. 2020 Oct 15;11:5206. doi: 10.1038/s41467-020-18871-1 (PMC7562722; doi:10.1038/s41467-020-18871-1)
Supplement: Supplementary file 5 — Reporting Summary [file 41467_2020_18871_MOESM5_ESM.pdf]

nature research

Corresponding author(s): Ohad Manor

Last updated by author(s): Aug 27, 2020

Reporting Summary

Nature Research wishes to improve the reproducibility of the work that we publish. This form provides structure for consistency and transparency in reporting. For further information on Nature Research policies, see Authors & Referees and the Editorial Policy Checklist.

Statistics

For all statistical analyses, confirm that the following items are present in the figure legend, table legend, main text, or Methods section.

n/a	Confirmed	
☐	☒	The exact sample size (n) for each experimental group/condition, given as a discrete number and unit of measurement
☐	☒	A statement on whether measurements were taken from distinct samples or whether the same sample was measured repeatedly
☐	☒	The statistical test(s) used AND whether they are one- or two-sided<br>*Only common tests should be described solely by name; describe more complex techniques in the Methods section.*
☐	☒	A description of all covariates tested
☐	☒	A description of any assumptions or corrections, such as tests of normality and adjustment for multiple comparisons
☐	☒	A full description of the statistical parameters including central tendency (e.g. means) or other basic estimates (e.g. regression coefficient) AND variation (e.g. standard deviation) or associated estimates of uncertainty (e.g. confidence intervals)
☐	☒	For null hypothesis testing, the test statistic (e.g. F, t, r) with confidence intervals, effect sizes, degrees of freedom and P value noted<br>*Give P values as exact values whenever suitable.*
☒	☐	For Bayesian analysis, information on the choice of priors and Markov chain Monte Carlo settings
☒	☐	For hierarchical and complex designs, identification of the appropriate level for tests and full reporting of outcomes
☐	☒	Estimates of effect sizes (e.g. Cohen's d, Pearson's r), indicating how they were calculated

Our web collection on statistics for biologists contains articles on many of the points above.

Software and code

Policy information about availability of computer code

Data collection	No software was used to collect the data. For lifestyle/diet/activity questions, the data was collected via a custom website built for this purpose.
Data analysis	Microbiome data was analyzed using the QIIME pipeline (version 1.9.1; default parameters). Functional inference was done using PICRUSt (version 1.0.0; default parameters). Statistical analysis and plotting was done in R version 3.5.1 (2018-07-02).

For manuscripts utilizing custom algorithms or software that are central to the research but not yet described in published literature, software must be made available to editors/reviewers. We strongly encourage code deposition in a community repository (e.g. GitHub). See the Nature Research guidelines for submitting code & software for further information.

Data

Policy information about availability of data

All manuscripts must include a data availability statement. This statement should provide the following information, where applicable:

- Accession codes, unique identifiers, or web links for publicly available datasets
- A list of figures that have associated raw data
- A description of any restrictions on data availability

The Institute for Systems Biology manages data requests for non-profit research purposes and will grant access to qualified researchers. Data requests should be sent to: andrew.magis@isbscience.org. Raw microbiome data has been previously made available under SRA accession SRP148278 [https://www.ncbi.nlm.nih.gov/sra/?term=SRP148278]. Closed-reference OTU picking against the Greengenes database (version 13_08, https://greengenes.secondgenome.com/). KEGG database was downloaded on July 3rd, 2017 (https://www.kegg.jp/kegg/download/).

Field-specific reporting

Please select the one below that is the best fit for your research. If you are not sure, read the appropriate sections before making your selection.

☒ Life sciences ☐ Behavioural & social sciences ☐ Ecological, evolutionary & environmental sciences

For a reference copy of the document with all sections, see nature.com/documents/nr-reporting-summary-flat.pdf

Life sciences study design

All studies must disclose on these points even when the disclosure is negative.

Sample size	We used all microbiome data available from previous members of the Arivale wellness program. Where available, we matched these data with clinical labs, digestion, diet, lifestyle, and activity data for the same individuals.
Data exclusions	We excluded samples from individuals who reported taking antibiotics in the last 3 months prior to microbiome sampling.
Replication	we did not replicate the experiment given that these were samples from individuals taken at a certain time.
Randomization	Samples were collected over a few years, and no intentional grouping was conducted.
Blinding	All data was de-identified and no information regarding grouping was available.

Reporting for specific materials, systems and methods

We require information from authors about some types of materials, experimental systems and methods used in many studies. Here, indicate whether each material, system or method listed is relevant to your study. If you are not sure if a list item applies to your research, read the appropriate section before selecting a response.

Materials & experimental systems

n/a	Involved in the study	
☒	☐	Antibodies
☒	☐	Eukaryotic cell lines
☒	☐	Palaeontology
☒	☐	Animals and other organisms
☐	☒	Human research participants
☒	☐	Clinical data

Methods

n/a	Involved in the study	
☒	☐	ChIP-seq
☒	☐	Flow cytometry
☒	☐	MRI-based neuroimaging

Human research participants

Policy information about studies involving human research participants

Population characteristics	Number of individuals: 3,028<br>Sex (% female): 59%<br>Age: 49±12<br>Ethnicity (% white): 84%<br>BMI: 27±6
Recruitment	All de-identified data are from consenting individuals in a commercially-available, non-medical lifestyle intervention program (Arivale Inc., Seattle, WA). Only data from individuals who provided explicit informed consent and authorization for their anonymized data for research use are included in this study. Data was collected starting in July 2015. The Arivale program involved health coaching on exercise, nutrition, stress management, and other wellness goals. Only baseline data prior to intervention from a total of 3,409 individuals who reported not taking antibiotics 3 months prior to sampling were included in the current study. This research project was performed independent of Arivale's commercial operations and was done entirely using only de-identified data.
Ethics oversight	Procedures for the current study were run under the Western Institutional Review Board at Arivale Inc. (Seattle, WA) and at the Institute for Systems Biology (IRB Study Number 1178906 and 20170658, respectively).

Note that full information on the approval of the study protocol must also be provided in the manuscript.
